# Tumor-induced inflammation alters neutrophil phenotype and disease progression

**DOI:** 10.1186/s13058-015-0644-6

**Published:** 2015-10-06

**Authors:** Charaf Benarafa

**Affiliations:** Theodor Kocher Institute, University of Bern, Freiestrasse 1, CH-3012 Bern, Switzerland

## Abstract

Neutrophils are essential to combat infectious agents but contribute to collateral inflammatory damage. Likewise, neutrophils can kill cancer cells and have been shown to promote malignant growth and metastasis through immunosuppressive functions. Two articles in a recent issue of *Nature* reveal new mechanisms by which tumors induce changes in neutrophil phenotype through production of inflammatory cytokines. Although the two studies report different outcomes on the effects of neutrophils on tumor growth and metastasis, they delineate novel molecular pathways influencing neutrophil phenotype that may provide new approaches to harnessing neutrophil functions in the treatment of cancer.

Neutrophils develop in the bone marrow and rapidly respond to danger signals by a prompt mobilization to injured tissues, where they accumulate, amplify inflammatory responses, eliminate pathogens, and sometimes induce local tissue injury. Recent studies indicate that neutrophils can acquire phenotypic changes with markedly altered functions and increased survival [1]. In cancer patients and tumor-bearing mice, a major question has been the elusive origin and functional characterization of tumor-associated neutrophils and granulocytic myeloid-derived suppressor cells (G-MDSCs), which can be considered a subset of neutrophils with immunosuppressive activity on T cells. In two recent studies published in *Nature* [2, 3], tumor-induced mechanisms were shown to alter neutrophil numbers and function, highlighting the plasticity of the neutrophil and its importance in controlling growth and invasiveness of cancer cells.

Several types of tumors are dependent on the activation of the tyrosine kinase receptor MET pathway. In the first highlighted article, Finisguerra et al. investigated the function of the *MET* proto-oncogene in stromal cells, including leukocytes associated with tumors [2]. They convincingly demonstrated that deletion of MET in neutrophils was associated with increased tumor growth and metastasis in multiple tumor models in mice, including spontaneous mammary tumors driven by transgenic expression of the polyoma virus middle T (PyMT) antigen. Tumor necrosis factor-alpha (TNF-α) and other soluble factors produced by tumor cells were responsible for *Met* induction in a subset of circulating neutrophils of tumor-bearing mice, and the Met^+^ neutrophil subset was enriched within the tumor mass and contributed to reduced tumor growth and metastasis. Mechanistically, the transmigration of anti-tumoral Met^+^ neutrophils was dependent on expression of high levels of hepatocyte growth factor (HGF), the only ligand for MET, by the tumor. It remains unclear why the transmigration of Met^+^ neutrophils in vivo was dependent on HGF produced in the tumor environment when these neutrophils could potentially respond to other chemotactic cues recruiting Met-negative neutrophils. Regardless, the investigators uncovered a potential flaw in MET targeting therapy in cancer, where the effects of MET kinase inhibitors, usually used to block tumor growth, are dampened by the inhibition of anti-tumoral neutrophils expressing *Met*.

Neutrophilia, or high neutrophil numbers in the circulation, is a common observation in tumor-bearing mice. Moreover, a high neutrophil-to-lymphocyte ratio in patients with solid tumors is associated with poor overall survival [4]. In the second highlighted article, Coffelt et al. investigated the mechanisms leading to the generation of large numbers of pro-metastatic immunosuppressive neutrophils in a single model of breast cancer mediated by combined deletion of p53 and E-cadherin [3]. They found that tumor-induced production of the pro-inflammatory cytokine interleukin (IL)-1β hijacks a previously described homeostatic cascade that promotes granulopoiesis by inducing IL-17 and granulocyte-colony-stimulating factor (G-CSF) [5]. Interestingly, depletion of immunosuppressive neutrophils and γδ T cells particularly impaired early metastatic spread but had little effect on the primary tumor growth. The pro-metastatic function of neutrophils was mediated by immunosuppression of CD8 cytotoxic T cells. Co-depletion of neutrophils and CD8-positive cells reverted the anti-metastatic phenotype associated with neutrophil depletion. It will be interesting to find out whether these findings are reproduced in other models.

The two studies raise several issues about harnessing inflammatory cytokines in cancer therapy. First, repurposing anti-inflammatory drugs and, more specifically, antibodies blocking inflammatory cytokines that act upstream of G-CSF could be envisaged for reducing neutrophil production. Indeed, G-CSF was shown to be necessary and sufficient to alter hematopoiesis in favor of production of immunosuppressive neutrophils in the PyMT model [6]. However, it is also likely that tumor-induced inflammation concurrently promotes the production of neutrophils with anti-tumor activity, such as Met^+^ neutrophils. Therefore, the net effect of such therapies may be variable depending on the tumor type, the tissue, and the host response. Second, concomitant inflammatory diseases and environmental exposures, such as cigarette smoke, may affect breast cancer growth and metastasis through effects on neutrophil phenotypes. Knowledge of molecular pathways of neutrophil functions in cancer remains patchy. For example, claims were made for the effects of inducible nitric oxide synthase for both pro- and anti-tumoral neutrophils in the two highlighted articles. This apparent contradiction may be due to the fact that the same neutrophil subset carries out both functions or that neutrophil subsets need to be better defined depending on the tumor and its microenvironment. Migratory and localization profiles of neutrophils within the primary tumor, endothelial surfaces, and pre-metastatic niches relative to tumor cells and T cells may tip the balance in different directions. In vivo live cell imaging of such interactions may be particularly revealing. In conclusion, these studies show that tumor-associated inflammation profoundly alters granulopoiesis and simultaneously releases neutrophils with different migratory and anti-tumor properties. The defined pathways highlighted here provide the basis for further studies to define intervention points to target tumor type-specific neutrophil migration and function.

## Abbreviations

G-CSF: Granulocyte-colony-stimulating factor
HGF: Hepatocyte growth factor
IL: Interleukin
PyMT: Polyoma virus middle T
